# Long-term effect of botulinum toxin A on the hip and spine in cerebral palsy: A national retrospective cohort study in Taiwan

**DOI:** 10.1371/journal.pone.0255143

**Published:** 2021-07-22

**Authors:** Ching-Yueh Lin, Chi-Hsiang Chung, Dennis J. Matthews, Heng-Yi Chu, Liang-Cheng Chen, Sung-Sen Yang, Wu-Chien Chien

**Affiliations:** 1 Department of Physical Medicine & Rehabilitation, Kaohsiung Armed Forces General Hospital, Kaohsiung, Taiwan; 2 School of Medicine, National Defense Medical Center, Taipei, Taiwan; 3 School of Public Health, National Defense Medical Center, Taipei, Taiwan; 4 Department of Medical Research, Tri-Service General Hospital, National Defense Medical Center, Taipei, Taiwan; 5 Taiwanese Injury Prevention and Safety Promotion Association, Taipei, Taiwan; 6 Department of Physical Medicine and Rehabilitation, Children’s Hospital Colorado, University of Colorado School of Medicine, Aurora, CO, United States of America; 7 Department of Physical Medicine and Rehabilitation, Tri-Service General Hospital, School of Medicine, National Defense Medical Center, Taipei, Taiwan; 8 Division of Nephrology, Department of Medicine, Tri-Service General Hospital, Graduate Institute of Medical Sciences, National Defense Medical Center, Taipei, Taiwan; National Yang-Ming University, TAIWAN

## Abstract

**Objectives:**

To investigate the effect of botulinum toxin A (BTA) on the development of hip dislocation and scoliosis, surgical rates for hip and spine, and mortality in cerebral palsy (CP).

**Study design:**

A cohort study was conducted using CP data from a Taiwan National Insurance Health Research Database. Diagnoses were defined using the International Classification of Diseases codes, 9th revision. Adjusted hazard ratios for outcomes were calculated using Cox regression analysis and adjusted for the following variables: BTA injection, sex, age, severities of CP, comorbidities, location, urbanization level, and level of care.

**Results:**

A total of 1,405 CP children (670 female vs. 735 male), 281 in the BTA group and 1,124 in the controls, were followed-up for a mean of 5 years 4 months. There were no significant differences in the outcomes in both groups, in the incidence rates of hip dislocation and scoliosis, nor in the surgical rates for hip and spine surgery. Mortality rate in the BTA group was 0.49 times lower than that in the controls (*p* = 0.001). Moderate to severe types of CP had higher incidence rates of hip dislocation, scoliosis, hip surgery, spine surgery, and mortality.

**Conclusion:**

Moderate to severe types of CP had poorer outcomes in all aspects, including a higher risk of hip dislocation, scoliosis, surgical rate for hip and spine, and mortality. Although BTA injection in children with CP proved to not significantly reduce hip dislocation and scoliosis, it is considered safe as an anti-spasticity treatment and may be beneficial for survival.

## Introduction

Cerebral palsy (CP) is not a rare disease in children, with its prevalence ranging from 2–3.5 per 1,000 livebirths and remained constant worldwide during the past four decades [1]. CP usually causes variable degrees of physical and mental disabilities, which in turn lead to substantial medical care costs and expenditure of social resources. Physical impairments include weakness, spasticity, dystonia, ataxia, epilepsy, and intellectual disability, among others. Use of botulinum toxin A (BTA) is gradually becoming popular because it can focally block spasticity, leading to a reduction in limb contracture and skeletal deformity without generating systemic side effects [2].

Several authors have been advocating the idea that administrating BTA in CP children with spasticity may alter the process of hip dislocation, but the results were inconsistent [3–8]. Different study designs, discrepancies in the case numbers, and variable durations of follow-up contributed to this inconsistency. A systemic review by Stacey claimed that there was lack of conclusive evidence regarding the effect of BTA injection or other conventional therapy on slowing or reducing hip displacement [9]. Only two studies have examined the effect of BTA on CP scoliosis, but unfortunately, their conclusions were inconsistent [10, 11]. The mortality in children with CP was higher than the normal population [12]. Severe motor impairment and cognitive impairment accounted for higher mortality [1, 12]. Respiratory disease was the leading cause of death in both childhood [12, 13] and adult stages of CP [14].

Due to the aforementioned controversies, we hypothesized that BTA was effective against hip dislocation and scoliosis in CP, and may subsequently reduce surgical rates for the hip and spine as well. We also hypothesized it had a beneficial effect on survival in children with CP. Additionally, we wanted to investigate the common causes of death in CP by using the National Health Insurance Research Database.

## Methods

### Demographic data

The National Health Insurance program, which was established in 1995, covered the health expenses of nearly 99.99% of 23 million residents in Taiwan. The data used in the study is a subset composed of 2 million people randomly collected from the original database of National Health Insurance program beneficiaries. There were no significant differences in age, sex, and income between the subset and the original database, and its quality and validity were verified by previous studies [15]. Demographic data, including sex, age, residential location, level of urbanization, and level of care were derived from the registry for the beneficiaries’ files. Our subjects were to discover how BTA affects musculoskeletal development, and find its relationship with mortality in children with CP. The study period was assigned from 01.01.2000 to 12.31.2015. Diagnoses and comorbidities were defined using the corresponding codes of the International Classification of Diseases, 9th Revision, Clinical Modification (ICD-9-CM) (S1 Table). The study design was approved by the institutional review board of Kaohsiung Armed Forces General Hospital (KAFGHIRB109-016), which exempted requirement for informed consent because of the use of de-identified patient data.

### Inclusion and exclusion criteria

**Fig 1:**
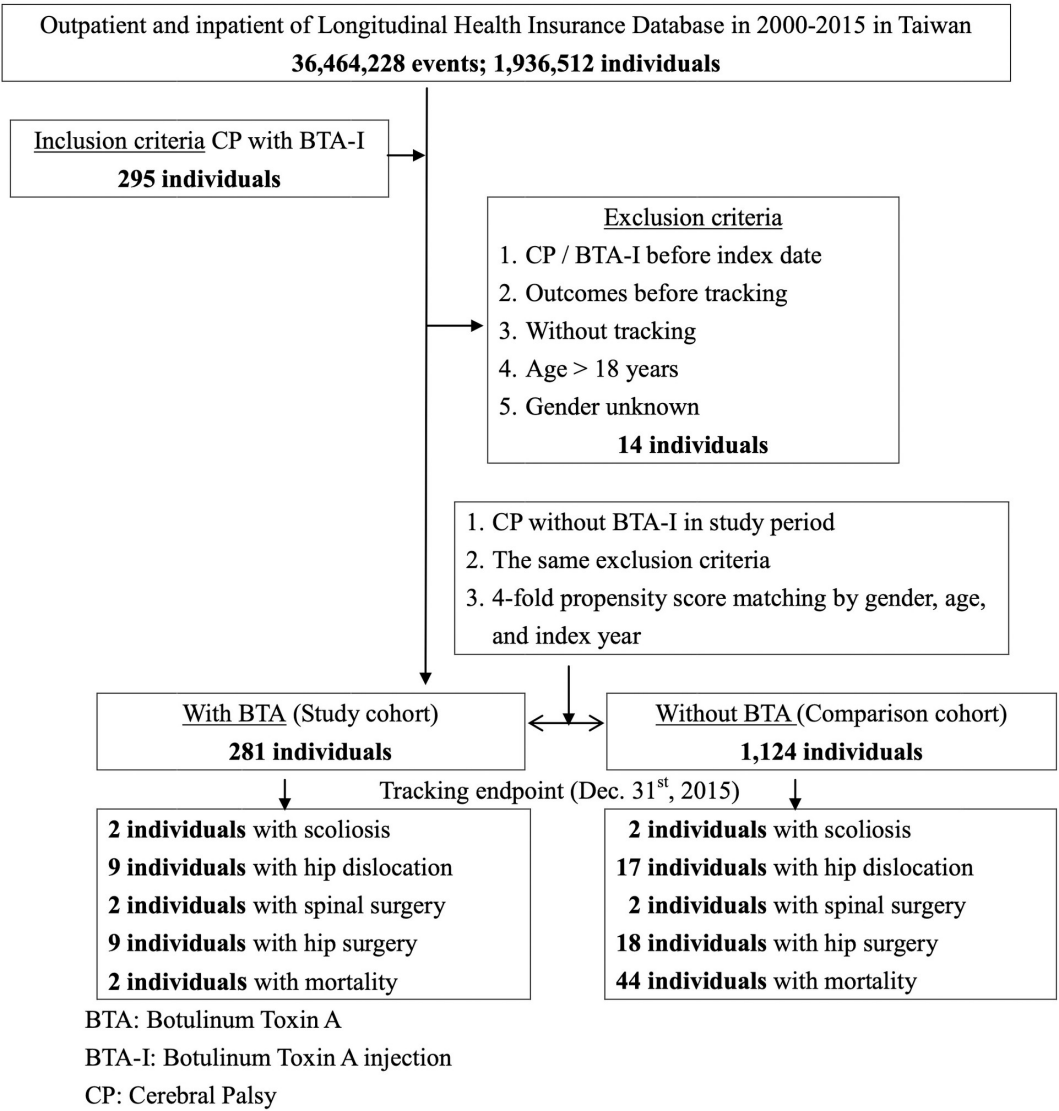
Flowchart.

In Fig 1, children with CP were defined as those who had at least three outpatient visits or one hospitalization recorded with ICD-9-CM codes 343.x. Moderate to severe CP was defined as having both a CP diagnosis and a catastrophic illness certificate. In Taiwan, people suffering from moderate to severe physical or mental disabilities are qualified for issuance of a catastrophic illness certificate based on the discretion and clinical judgment of a specialist. Subjects older than 18 years old, of unknown sex, or those with any hip or spine-related event prior to the diagnosis of CP were excluded. Children with a diagnosis of CP who received BTA injections during the study period were allocated to the BTA group, while those who did not receive BTA injections within the study period were allocated to the non-BTA group. Propensity score matching was performed using a ratio of 1:4 for the treatment and control groups, wherein match tolerance was set at 0.15. The propensity score matching was set as using logistic regression in estimation algorithm and nearest neighbor matching in matching algorithm. The options for nearest neighbor were random in matching order, non-replacement, 1 to 4 matching, and no caliper. Covariates such as sex, age, and index date were balanced. Index dates for the BTA group was set as the day the children with CP had their first BTA injection, while for the non-BTA group, the index date was set as the day when the children with CP who had not received any BTA injection were enrolled using the matching method. The end points were set as the day mortality occurred or the end of the study period.

### Intervention

In accordance with FDA regulations, Taiwanese physicians practice BTA injection as early as 2 years of age in CP children when they have spasticity. There is no upper limit for the age wherein BTA injection is allowed. During the study period, there were two available BTA medications: Botox (Allergan Pharmaceuticals Ireland, Westport County Mayo, Ireland) and Dysport (Ipsen Biopharm Limited, Berkshire, UK), for treating spasticity in CP in Taiwan. BTA injections were defined by a procedure code. Anti-spasticity blocks were combined with a drug code of either Botox or Dysport (S1 Table).

### Outcome

The primary outcomes in this study were hip dislocation or scoliosis related to CP at any age, as well as having any surgical intervention for hip dislocation or scoliosis. Hip dislocation included any types of dysplasia or dislocation of the hip. Hip surgery was defined as any bony and soft tissue orthopedic surgery for the hip. Spine surgery was defined as the use of various fusion techniques for scoliosis. Corresponding ICD-9-CM codes are listed in S1 Table.

The secondary outcome in this study was mortality from CP.

### Comorbidities

Comorbidities, including intellectual disability, dystonia, epilepsy, and short gestation with low birthweight were used to analyze any additional risk for developing hip dislocation or scoliosis, the need for surgical intervention, and mortality (S1 Table).

#### Statistical analysis

SPSS software version 22 was used for all analyses in this study. A Chi-square test and Fisher exact test were used to examine categorical variables, including sex, age groups, severities of impairment, aforementioned comorbidities, location, urbanization level, and level of care. Age was divided into subgroups (0–2, 3–4, 5–6, 7–8, 9–10, 11–12, ≥13 years) for stratification. Cox regression for multivariate analysis was used to further determine the adjusted hazard ratio with a 95% confidence interval for the following variables: BTA injection, sex, age groups, severities of impairment, aforementioned comorbidities, location, urbanization level, and level of care. The proportional hazard assumption in Cox regression model was not violated as Global test P = 0.8497 was obtained using proportional hazard assumption test based on Schoenfeld residuals. Proportional hazard assumption is violated if Global test P < 0.05 [16]. The Kaplan-Meier method with a log-rank test was used to determine the cumulative risk of incidence of outcomes and survival among individuals with CP stratified by BTA injection. A *p-*value < 0.05 was considered statistically significant.

## Results

At baseline (Fig 1 and Table 1), from the 1,405 children with CP during 2000–2015 that were included in the current study, 281 cases had received BTA injections and 1,124 had not. They were followed-up for a mean of 5 years and 4 months (Table 2). The number of female and male CP patients were 670 and 735, respectively, with a slight male predominance having no significant intergroup difference. Children with CP were diagnosed between 0–10 years old, majority of which were diagnosed before the age of 2 years. The prevalence of dystonia and epilepsy were significantly higher in the control group both at baseline and at follow-up. The ratio of intellectual disability was initially higher in the control group, but the difference was not significant at follow-up. The rates of short gestation with low birthweight in both groups were similar (32/281, 11.39% vs. 134/1124, 11.92%, data collected on the date of birth). The ratio of moderate to severe type CP in both groups were also comparable (211/281, 75.1% vs. 838/1124, 74.6%). More children with CP were registered in places with higher urbanization level (*p* = 0.07), in northern Taiwan (*p* = 0.011), and in hospital centers (*p* = 0.003).

**Table 1:** Characteristics of the study population at baseline.

CP	Total		BTA		Non-BTA		*p*
Variables	n	%	n	%	n	%	
Total	1,405		281	20.00	1,124	80.00	
Gender							0.999
Male	735	52.31	147	52.31	588	52.31	
Female	670	47.69	134	47.69	536	47.69	
Age (years)	2.53 ± 1.89		2.50 ± 1.81		2.53 ± 1.91		0.794
Age group (years)							0.999
0–2	927	65.98	201	71.53	726	64.59	
3–4	378	26.90	60	21.35	318	28.29	
5–6	50	3.56	10	3.56	40	3.56	
7–8	40	2.85	8	2.85	32	2.85	
9–10	10	0.71	2	0.71	8	0.71	
11–12	0	0	0	0	0	0	
≧13	0	0	0	0	0	0	
Catastrophic illness	1,049	74.66	211	75.09	838	74.56	0.854
Intellectual disability	9	0.64	0	0.00	9	0.80	0.003
Dystonia	6	0.43	0	0.00	6	0.53	0.006
Epilepsy	223	15.87	11	3.91	212	18.86	<0.001
Short gestation with low birthweight	3	0.21	0	0.00	3	0.27	0.012
Location							0.011
Northern Taiwan	667	47.44	131	46.45	536	47.69	
Middle Taiwan	360	25.60	89	31.56	271	24.11	
Southern Taiwan	287	20.41	53	18.79	234	20.82	
Eastern Taiwan	92	6.54	9	3.19	83	7.38	
Outlets islands	0	0.00	0	0.00	0	0.00	
Urbanization level							0.071
1 (The highest)	669	47.62	151	53.74	518	46.09	
2	537	38.22	90	32.03	447	39.77	
3	34	2.42	5	1.78	29	2.58	
4 (The lowest)	165	11.74	35	12.46	130	11.57	
Level of care							0.003
Hospital center	854	60.78	187	66.55	667	59.34	
Regional hospital	468	33.31	71	25.27	397	35.32	
Local hospital	83	5.91	23	8.19	60	5.34	

Chi-square / Fisher exact test on category variables and t-test on continue variables

BTA = Botulinum toxin A, CP = cerebral palsy

**Table 2:** Years of follow-up.

BTA	Min	Median	Max	Mean ± SD
With	0.01	4.78	15.68	6.42 ± 3.84
Without	0.01	3.12	15.77	4.98 ± 4.22
Total	0.01	4.09	15.77	5.36 ± 4.15

BTA = Botulinum toxin A, SD = standard deviation

During the follow-up (Table 3), a higher ratio of hip dislocation (3.2% vs. 1.6%) and hip surgery (3.2% vs. 1.6%) as well as a higher ratio of scoliosis (0.71% vs. 0.18%) and spine surgery (0.71% vs. 0.18%) were noted in the BTA group; however, there were no significant differences compared to controls. There was significant lower mortality in the BTA group (0.71% vs. 2.85%, p = 0.037).

**Table 3:** Characteristics of the study population at the endpoint.

CP	Total		BTA		Non-BTA		*p*
Variables	n	%	n	%	N	%	
Total	1,405		281	20.00	1,124	80.00	
Scoliosis	4	0.28	2	0.71	2	0.18	0.181
Hip dislocation	26	1.85	9	3.20	17	1.51	0.079
Spinal surgery	4	0.28	2	0.71	2	0.18	0.181
Hip surgery	27	1.92	9	3.20	18	1.60	0.090
Mortality	34	2.42	2	0.71	32	2.85	0.037

Chi-square / Fisher exact test on category variables and t-test on continue variables

BTA = Botulinum toxin A, CP = cerebral palsy

**Fig 2:**
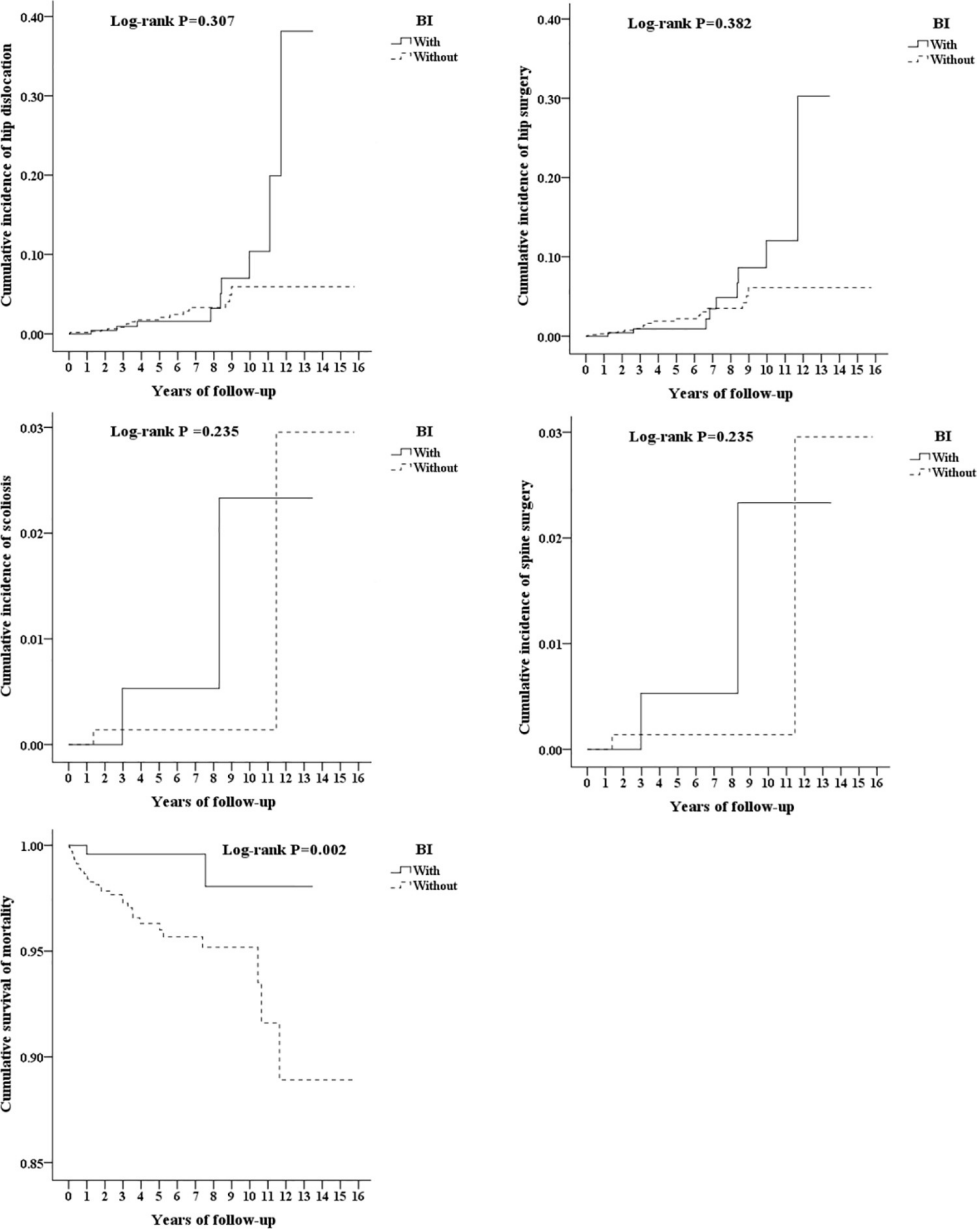
Cumulative incidence of outcomes. Top left: hip dislocation. Top right: hip surgery. Middle left: scoliosis. Middle right: spine surgery. Bottom left: cumulative survival of mortality.

Upon Cox-regression analysis (Table 4), there was no significant difference seen in terms of developing hip dislocation or scoliosis based on BTA was given or not. Mortality rates in the intervention group were 0.49 times lower compared to controls (*p* = 0.001). The cumulative incidence of outcomes was shown in Fig 2. Neither sex nor the presence of any comorbidities had significant influence on the outcomes. Moderate to severe CP has a 2.3-fold increased risk of hip dislocation and a 2.6-fold increased risk of scoliosis, a 2.1-fold increased rate of hip surgery and a 2.6-fold increased rate of spine surgery, and a 1.9-fold increased risk of mortality as mild CP. Majority of the subjects who had hip dislocation had developed it at a very young age of 3–4 years old. Hip surgery was undertaken at about the same time. Scoliosis and spinal surgery were more commonly done at age 9–10 and 11–12. Age was significantly inversely related to mortality rate (*p* < 0.001). The development of hip dislocation or scoliosis, the time for hip or spine surgery, and mortality were all encountered at older ages in the BTA group. Respiratory diseases (23/34 [67.6%]) were found to be the main cause of death in CP, with pneumonia being the most common among them (12/34 (35.3%)) (Table 5).

**Table 4:** Factors of outcomes after Cox regression analysis.

Outcomes		Scoliosis				Hip dislocation				Spinal fusion				Hip revision surgery				Mortality			
Variables		Adjusted HR	95% CI	95% CI	*P*	Adjusted HR	95% CI	95% CI	*P*	Adjusted HR	95% CI	95% CI	*P*	Adjusted HR	95% CI	95% CI	*P*	Adjusted HR	95% CI	95% CI	*P*
BTA		2.849	0.304	26.745	0.358	1.263	0.530	3.007	0.595	2.849	0.304	26.745	0.358	1.198	0.496	2.893	0.685	0.490	0.067	0.866	0.001
Gender		1.205	0.133	10.943	0.866	0.793	0.353	1.779	0.575	1.205	0.133	10.943	0.866	0.713	0.322	1.577	0.405	1.037	0.504	2.133	0.917
Age (yrs)		0.078	0.001	5.124	0.862	0.876	0.042	3.889	0.284	0.078	0.001	5.124	0.862	0.295	0.086	0.994	0.047	0.131	0.011	0.386	<0.001
Age (yrs)	0–2	Reference				Reference				Reference				Reference				Reference			
	3–4	-	-	-	-	6.793	0.635	72.619	0.113	-	-	-	-	1.255	0.248	6.347	0.781	0.300	0.114	0.792	0.015
	5–6	-	-	-	-	3.254	0.274	38.589	0.349	-	-	-	-	0.777	0.135	4.481	0.778	0.131	0.038	0.447	0.001
	7–8	-	-	-	-	2.382	0.221	25.670	0.473	-	-	-	-	0.388	0.069	2.201	0.286	0.022	0.002	0.193	0.001
	9–10	0.058	0.001	3.997	0.187	0.274	0.021	3.502	0.319	0.058	0.001	3.997	0.187	0.107	0.017	0.684	0.018	-	-	-	-
	11–12	0.075	0.001	7.313	0.267	0.413	0.036	4.711	0.477	0.075	0.001	7.313	0.267	0.123	0.020	0.778	0.026	0.023	0.003	0.158	<0.001
	≧13	-	-	-	-	0.187	0.017	2.105	0.175	-	-	-	-	0.072	0.012	0.438	0.004	0.004	0.000	0.055	<0.001
Catastrophic illness		2.575	1.049	3.450	0.001	2.311	1.040	3.326	0.010	2.575	1.049	3.450	0.001	2.126	1.037	3.000	0.014	1.896	1.025	2.264	0.028
Mental retardation		0.004	0.001	8.490	0.992	1.000	0.519	8.026	0.944	0.004	0.001	8.490	0.992	0.985	0.340	6.334	0.911	1.119	0.784	4.202	0.872
Dystonia		0.003	0.001	1.296	0.995	-	-	-	-	0.003	0.001	1.296	0.995	-	-	-	-	-	-	-	-
Epilepsy		0.002	0.001	1.157	0.869	0.448	0.151	1.332	0.149	0.002	0.001	1.157	0.869	0.394	0.133	1.164	0.092	0.469	0.186	1.184	0.110
Short gestation with low birthweight		-	-	-	-	-	-	-	-	-	-	-	-	-	-	-	-	-	-	-	-

BTA = Botulinum toxin A, CI = confidence interval, HR = hazard ratio.

Adjusted HR: Adjusted variables listed in the table

-: it means that the case number was zero in either of the two groups in the Cox regression analysis, so 95% CI, adjusted HR and P-value were not available.

**Table 5:** Distribution of the causes of death in both groups.

ICD-CM-9	BTA(n)	Non-BTA(n)	Total
486 Pneumonia^§^	0	12	12
485 Bronchopneumonia^§^	1	3	4
507.0 Aspiration pneumonitis^§^	0	2	2
5188 Respiratory failure^§^	0	5	5
0389 Septicemia	0	2	2
72781 Contracture of tendon (sheath)	1		1
155.0 Malignancy of liver	0	1	1
277.2 Other disorders of purine and pyrimidine metabolism	0	1	1
322.9 Meningitis	0	1	1
427.5 Cardiac arrest	0	1	1
474.12 Hypertrophy of adenoids alone	0	1	1
478.74 Stenosis of larynx	0	1	1
599.0 Urinary tract infection	0	1	1
785.59 Non-traumatic shock	0	1	1
Total	2	32	34

BTA = Botulinum toxin A, n = number

^§^Respiratory diseases

## Discussion

To investigate the long-term effect of BTA on CP, we retrospectively enrolled and studied a large population of children with CP from a Taiwan Health Insurance Research Database, for which the mean duration of follow-up was 5 years and 4 months. There was no significant difference between the intervention and control group with regards to age, sex, and disease severity. Both groups had around 75% having moderate to severe CP and 25% having mild CP that remained constant throughout the study period. Dystonia, epilepsy, and short gestation with low birthweight had no correlation with any of the outcomes.

GMFCS (Gross Motor Function Classification System) is a five-level scale for children with CP, and is based on gross motor functionality and the need for assistive device and wheeled mobility [17]. We defined children with CP who had a catastrophic card as having moderate to severe CP needing further assistive device or wheelchair for activities and ambulation, which resemble those in GMFCS III-V. From the current study’s results, moderate to severe CP patients had higher rates of scoliosis, hip dislocation, and mortality. It can be explained that more spasticity and lack of ambulation led these kids to be at higher risk for hip dislocation and scoliosis [18–22]. Previous studies also find severe motor impairment to be related to poor survival [12, 23]. Himmelmman found that higher mortality in CP was seen in those with spastic tetraplegia or dyskinesia, severe motor impairment, or cognitive impairment [12]. Glinianaia reported that having three or more severe impairments in the upper limb, lower limb, intellect, hearing, and vision were associated with significantly higher mortality in childhood CP [23].

Current therapies to reduce hip dislocation or hip surgery are variable, and include positioning, BTA with or without bracing, obturator nerve block, intrathecal baclofen pump, selective dorsal rhizotomy, or complementary and alternative medicine; however, the evidence of their efficacies were low [9]. Jung reported a promising effect of regular BTA injection on 27 children with CP after a 2-year duration to keep their hips stable [6]. In contrast, Graham found no preventive effect of regular BTA injection with hip bracing on hip dislocation for children with CP in the long term [4, 8]. Jung [6] claimed that more frequent BTA injection, different medication, and patient selection led to their results, but the shorter follow-up duration compared with Graham’s studies [4, 8] made their results inconclusive. Boyd firstly attempted combining BTA injection and bracing in children with CP, which suggested no superior therapeutic effect on gross motor function [3]. Graham indicated that BTA injection with bracing may be effective against hip contracture but not hip dysplasia and hip dislocation in the long term [4, 8]. The finding was explained by the fact that hip dysplasia and hip displacement were largely caused by lack of functional ambulation rather than adductor spasticity [4, 8]. Yang claimed that BTA injection was as effective as soft tissue surgery in preventing of hip dislocation; however, but there was no control group [5]. In our study, we found no significant difference in hip dislocation rate whether BTA was administered or not. Based on previous studies and the current study’s results, BTA injection alone did not prove to be an effective preventive strategy for hip dislocation.

Scoliosis usually happens secondary to hip dislocation, leading to deterioration of muscle forces and ambulation ability in CP [18–20, 22]. Some studies tried employing neurolytic blocking of back muscles to reduce muscle imbalance-related spinal curve progression in CP patients. Nuzzo demonstrated that the short-term effect of BTA injection can be a bridge for halting spinal curve progression before surgery [10]. Wong stated that BTA had no long-term clinical effect on scoliosis [11]. Our result also showed that BTA injection alone was not effective in the prevention of scoliosis in the long-term.

Interestingly, we noticed a significant lower mortality in the BTA group. BTA can be used to effectively reduce spasticity and pain [24–26], reduce drooling [27], improve motor functionality [26], ease provision of care, and increase quality of life [24]. Adverse effects of BTA are common, but are usually mild and self-limiting [28, 29]. All these factors may lead to better survival for CP patients, just as what our results showed. Furthermore, we found that respiratory disease, particularly pneumonia, to be a leading cause to mortality in children with CP (Table 5). Himmelmman [12] and Prastiya [13] had reported that aspiration and pneumonia accounted for a large portion of deaths in childhood CP. Ryan also found a 14-fold increased risk of death related to respiratory diseases in adult CP [14].

Despite the current study finding BTA alone to not be an effective preventive method for hip dislocation and scoliosis, there are still plenty of ways to improve musculoskeletal development in CP. In Sweden, a CP population health care program had been implemented for decades. They shared success in using the program for early detection and early treatment of spasticity, contractures, and hip migration, thus eventually reducing the need for orthopedic surgery in children with CP [2]. That encourages us and other countries to learn and develop our own CP surveillance programs. From our results, children with CP treated in hospital centers had a higher chance of receiving BTA injection (data no shown), which is consistent with the policy of the National Health Insurance that only physicians in a regional hospital or hospital center can claim reimbursement for BTA treatment. In the near future, optimal referral of children in need of BTA injection to a specialized hospital is also warranted.

The current study’s strength is its use of a nationwide database, which provided a larger population with less bias for sample selection, ethnics, religious, and geographic factors, and a longer observation duration compared to other studies. However, the study may be biased by the confounding by indication. While our result disclosed an insignificant effect of BTA on CP children in prevention of scoliosis, it still has a probability of type II error. We will need a larger study population to address the concern. Further, the limited study period and follow-up duration curbed the understanding of a lifelong picture of the effects of BTA on older children and adult CP. Lastly, details on functional classification, such as GMFCS, impaired region, protocols of BTA administration (treatment locations, intervals, and dosages), and any add-on treatments were not acquired from the database.

## Conclusions

In our study, moderate to severe types of CP had poorer outcomes in all aspects, including having a higher risk for hip dislocation, scoliosis, surgical rates for hip and spine, and mortality. Although BTA injection in children with CP did not lead to significant reduction of hip dislocation and scoliosis, it is safe as an anti-spasticity treatment and may be beneficial for survival.

## Supporting information

S1 Table ICD-9-CM, procedure codes, and drug codes. (DOCX)

## Abbreviations

BTA: botulinum toxin A
CP: cerebral palsy
ICD-9-CM: International Classification of Diseases, 9th Revision, Clinical Modification
