# Supplementary material for: Long-term effect of botulinum toxin A on the hip and spine in cerebral palsy: A national retrospective cohort study in Taiwan
Source: PLoS One. 2021 Jul 22;16(7):e0255143. doi: 10.1371/journal.pone.0255143 (PMC8297884; doi:10.1371/journal.pone.0255143)
Supplement: S1 Table — (DOCX) [file pone.0255143.s001.docx]

S1 Table. ICD-9-CM, procedure codes, and drug codes.

| Diagnosis (definition) | ICD-9-CM |
| --- | --- |
| Stduy population: Cerebral palsy | 343; Outpatinet ≧3 visits or inpatient |
| Intervention: Anti-spasticity block | 41005C; Botox, Dysport |
| Outcomes |  |
| Scoliosis | 737.43 |
| Hip dislocation (any type of dysplasia or dislocation of hip) | 718.75, 754.3, 835.0 |
| Spine surgery (different fusion technique for spine) | OP81.00, OP81.04, OP81.05, OP81.06, OP81.07, OP81.08; combined with 737.43 |
| Hip surgery (different bony and soft-tissue surgery for hip) | OP77.25, OP77.35, OP77.75, OP77.85, OP78.25, OP78.35, OP78.45, OP79.85, OP80.95, OP81.21, OP81.40, OP81.51-OP81.52, OP83.12; combined with 718.75, 754.3, 835.0 |
| Mortality |  |
| Comorbidities |  |
| Intellectual disability | 317-319 |
| Dystonia | 333 |
| Epilepsy | 345 |
| Short gestation with low  birthweight | 765 |
| Drug codes |  |
| Botox | K000525299, KC00525299, X000006255, X000017255 |
| Dysport | K000691299, K000870299, KC00870299 |
